# Ovarian Grafts 10 Days after Xenotransplantation: Folliculogenesis and Recovery of Viable Oocytes

**DOI:** 10.1371/journal.pone.0158109

**Published:** 2016-06-30

**Authors:** Paulo Henrique Almeida Campos-Junior, Thalys Jair Melo Alves, Marco Tulio Dias, Carolina Marinho Assunçao, Michele Munk, Matheus Silvério Mattos, Lucas Rocha Kraemer, Brígida Gomes Almeida, Remo Castro Russo, Lucíola Barcelos, Luiz Sérgio Almeida Camargo, Joao Henrique Moreira Viana

**Affiliations:** 1 Fertility Preservation Research Group, Department of Natural Sciences, Federal University of Sao Joao Del Rei, Sao Joao Del Rei, Minas Gerais, Brazil, 36301–160; 2 Laboratory of Genetics and Biotechnology, Department of Biology, Federal University of Juiz de Fora, Juiz de Fora, Minas Gerais, Brazil, 36036–900; 3 Department of Physiology and Biophysics, Institute of Biological Sciences, Federal University of Minas Gerais, Belo Horizonte, Minas Gerais, Brazil, 31270–901; 4 Embrapa, Juiz de Fora, Minas Gerais, Brazil, 36038–330; 5 Embrapa, Brasilia, Distrito Federal, Brazil, 70770–917; Queen's University, CANADA

## Abstract

Ovarian xenotransplantation is a promising alternative to preserve fertility of oncologic patients. However, several functional aspects of this procedure remained to be addressed. The aim of this study was evaluate the feasibility of xenotransplantation as a strategy to maintain bovine ovarian grafts and produce oocytes. Adult ovarian cortical pieces were xenotransplanted to the dorsal subcutaneous of female NOD-SCID mice (n = 62). Grafts were recovered ten days after xenotransplantation. Host and graft weights; folliculogenesis progression; blood perfusion, relative gene expression and number of macrophage and neutrophil of xenografts; *in vitro* developmental competence of graft-derived oocytes were evaluated. Folliculogenesis was supported in the grafts, as indicated by the presence of primordial, primary, secondary, antral, and atretic follicles. The xenografts showed a greater volumetric density of atretic follicles and higher hyperemia and number of host-derived macrophage and neutrophil (*P<*0.05), when compared to non-grafted fragments. There was a higher blood perfusion under the back skin in the transplantation sites of host animals than in control and non-grafted (*P<*0.01). *BAX* and *PRDX1* genes were up-regulated, while *BCL2*, *FSHR*, *IGF1R* and *IGF2R* were down-regulated, when compared to the control (*P<*0.01). Twenty seven oocytes were successfully harvested from grafts, and some of these oocytes were able to give rise to blastocysts after *in vitro* fertilization. However, cleavage and blastocyst rates of xenograft derived oocytes were lower than in control (*P<*0.01). Despite showing some functional modifications, the ovarian xenografts were able to support folliculogenesis and produce functional oocytes.

## Introduction

In the past few decades, advances in the treatment of oncologic conditions have led to considerable improvement in survival rate of patients [1,2]. Unfortunately, chemotherapy and radiotherapy often cause infertility by destruction of the ovarian reserve, and usually these patients require further hormone replacement therapy and eventually need to undergo assisted reproduction [2,3]. Few options are currently available to preserve their fertility, such as transposition of ovaries before radiotherapy, and embryo or oocyte cryopreservation [2]. A promising new alternative is the cryopreservation of ovarian pieces before oncologic treatment, which makes possible to store a large number of follicles. This procedure could be quickly performed at any time in the menstrual cycle, with no delay in the oncologic treatment, and is a unique option for preserving fertility in prepubertal and premenarcheal female patients [2]. To date, more than 60 live human births were reported after auto-transplantation of ovarian tissue [2], and the demand for this kind of procedure is expected to increase in a near future [3].

One of the main concerns related to auto-transplantation in oncologic patients is the risk of the re-introduction of malignant cells. In this regard, human ovarian tissue xenotransplantation/grafting into mice has proved to be a safe alternative, because cancer cells are not able to penetrate the zona pellucida of xenografted oocytes [3–7], however, there is a report in the literature that indicated that the global risk of transplanting malignant cells using autotransplantation is low [8]. On the other hand, this technique can be useful as a model for examining ovarian function, *in vivo* follicle development, and for improving cryopreservation and grafting protocols [2,6,7]. In a classic study, Snow *et al*. (2002) [9] reported that mouse ovarian follicles developed after xenografted under the kidney capsule of nude rats, and the recovered oocytes were matured, *in vitro* fertilized, and resulted in the birth of live pups. Afterwards, other studies showed the feasibility of human ovarian xenotransplantation [1], however, due to the ethical and logistic restrictions, the developmental competence of human xenograft-derived oocytes remained to be investigated. Alternatively, the bovine model has been used [10]. Senbon *et al*. (2005)[11] showed that despite many of xenograft derived oocytes developed up to the 5- to 8-cell stage, no embryo reached the blastocyst stage. Therefore, the developmental competence of oocytes derived from xenografts is still unknown.

To become a useful procedure, a better understanding of follicular dynamics in xenografted ovarian pieces is also required [7]. Most of the studies published on this issue focused on long term evaluations (weeks or months after xenotransplantation), however they were unanimous in claim that the initial days after grafting is a critical period for follicular survival and development [2,10,12]. Thus, the evaluation of functional changes in the initial period after transplantation may provide new clues on the fate of the grafts. Neovascularization, for instance, is a mandatory aspect for graft establishment and follicle survival [12]. Up to 5 days after transplantation, Van Eyck et al. (2010)[13] identified a hypoxic period; however, more studies should be performed to investigate the subsequent period. As this time point is critical to graft development, the balance in the expression of anti-apoptotic genes (B-cell lymphoma 2—*BCL2*) and pro-apoptotic genes (BCL2-Associated X Protein–*BAX*; [14]) and oxidative stress markers (peroxiredoxin-1—*PRDX1*; [15]), as well as the expression pattern of other genes used as markers of follicular function, such as follicle-stimulating hormone receptor (*FSHR)*, insulin-like growth factor 1 and 2 receptors (*IGF1R* and *IGF2R*; [16]) may also be important to evaluate the graft establishment and development. Thus, the aim of this study was to evaluate, using the bovine graft model and in a short time after xenotransplantation, several functional aspects of the grafts, such as (*i*) folliculogenesis maintenance, (*ii*) graft histological components, (*iii*) blood perfusion, (*iv*) gene expression, (*v*) number of host-derived macrophage and neutrophil, (*vi*) and developmental competence after *in vitro* fertilization of xenograft-derived oocytes.

## Material and Methods

Female non-obese diabetic/severe combined immunodeficiency (NOD/SCID) mice (n = 62; 6 weeks of age) were used in this study: xenograft host (n = 46), non-manipulated (n = 8; control group) and incised but non-grafted (n = 8; non-xenografted group). Mice were obtained from University of São Paulo and maintained in a positive pressure room, isolated from other animals. Cages were filter-topped, and the mice had free access to sterile food and water. The daily light cycle was 12/12 h (light/dark). Animals were inspected daily, euthanasia was performed using high anesthetic doses (15.8 mg/kg xylazine and 139.6 mg/kg ketamine), and no animal died prior to the experimental endpoint. The Institutional Animal Care and Use Committee at Federal University of Sao Joao Del Rei specifically approved this study (Protocol number 009/15—CEUA/FUSJ), in order to alleviate the animal post-surgery suffering, analgesic (Dipyrone, 50 mg/kg) was administered daily.

### Bovine Ovarian Tissue for Xenotransplantation

Fresh bovine ovarian cortical tissue was obtained from local slaughterhouse (Abatedouro Municipal de São Joao Del Rei, 21°08'09" S and 44° 15' 43"W), and both ovaries were recovered from each animal (graft donors, n = 8). Ovaries were immediately placed in Phosphate Buffer Saline—PBS (Gibco BRL; Life Technologies, Rockville, MD, USA) and transported at 25°C to the laboratory within 10 min. Ovarian cortical tissue samples were isolated and cut into small pieces of approximately 1.5 mm^3^. Some of these ovarian fragments were fixed (control for histology) or were frozen at -80°C (controls for gene expression analysis, macrophage and neutrophil counts).

### Xenotransplantation

Under laboratory conditions, ovarian pieces (n = 184) were washed three times with PBS, and maintained in supplemented DMEM on ice until xenograft implantation. To evaluate the donor ovary histological status before grafting, representative ovarian tissue fragments were fixed in Bouin’s solution and routinely processed for histological analysis. Female immunodeficient SCID mice (n = 46) were anesthetized (7.9 mg/kg xylazine and 69.8 mg/kg ketamine) as described elsewhere [17], and the dorsal area of each mouse was shaved and the skin aseptically cleaned immediately before surgery. Four ovarian pieces were then grafted into a small subcutaneous pocket under the back skin of each recipient mouse. Two incisions were made on each side of the dorsal midline, just above the fore and hind limbs, and they were sutured after transplantation [18].

### Biometrical and histological evaluations

Weights of the mice and ovarian pieces were measured before transplantation and after recovery, 10 days later. The grafts were then fixed in Bouin’s solution for approximately 24 h, placed in ethanol (70%) and stored at room temperature until they were embedded in paraffin. Embedded grafts were serially sliced (5 μm) for a total of 40 sections per graft. The slices were stained with hematoxylin/eosin and were qualitatively examined for the presence of ovarian follicles (primordial, primary, secondary, antral, and atretic), which were classified as previously described [19]. Additionally, the volume densities of the graft histological components were determined by images captured with light microscopy, using a 540-intersection grid from ImageJ software (National Institutes of Health, http://rsb.info.nih.gov/ij/). Fifteen randomly chosen fields/images (8,100 points) were scored per graft at 200X magnification [20]. In this analysis the following structures and parameters were considered: healthy follicles, atretic follicles, connective tissue, blood vessels, and hyperemia.

### Quantification of Neutrophil and Macrophage by Myeloperoxidase (MPO) and N-Acetylglucosaminidase (NAG) Activity

The MPO assay was performed, as previously described [21, 22], to estimate the number of neutrophils in xenografted and non-xenografted ovarian pieces. This assay employed 25 mL of 3,3’-5,5’-tetramethylbenzidine (TMB; Sigma Chemical Co., USA), diluted in dimethyl sulfoxide (DMSO; Merck, Darmstadt, Germany) at a final concentration of 1.6 mM, 100 mL of H_2_O_2_, diluted in phosphate buffer (pH 5.4) containing HTAB in a final concentration of 0.002% vol/vol and 25 μL of supernatant from tissue sample processed. The reaction was started at 37°C for 5 min in a 96-well microplate by adding the supernatant and the TMB solution. After that, H_2_O_2_ was added and followed by a new incubation at 37°C for 5 min. The reaction was stopped by adding 100 μL of 1M H_2_SO_4_ and samples were quantified at 450 nm in a spectrophotometer (Emax; Molecular Devices, Sunnyvale, CA, USA). Neutrophil number in each sample was calculated from a standard curve of neutrophils obtained from the peritoneal cavity of 5% casein-treated animals and processed in the same way. These results were expressed as relative number of neutrophils per mg of wet tissue.

The NAG assay was performed to evaluate the number of macrophages in xenografted and non-xenografted ovarian pieces [21, 22]. This reaction was started at 37°C for 10 min in a 96-well microplate by the addition of 100 μL of p-nitrophenyl-N-acetyl-D-glucosaminide (Sigma Chemical Co.), diluted in citrate/phosphate buffer (0.1 M citric acid, 0.1 MNa_2_HPO_4_, pH 4.5) in a final concentration of 2.24 mM/100 μL of supernatant from tissue sample processed, at appropriate dilutions in citrate/phosphate buffer. The reaction was finished by adding 100 μL of 0.2 M glycine buffer (pH 10.6) and samples were quantified at 405 nm in a spectrophotometer (Emax, Molecular Devices). The macrophage content was calculated from a standard curve based on NAG activity, which was expressed as the absorbance increase at 405 nm from 3% thioglycollate peritoneal-induced macrophages assayed in parallel. These results were expressed in relative number of macrophages (x 10^4^) per mg of wet tissue.

### Blood perfusion analysis

Blood perfusion analysis in the skin above the xenografts was carried out as previously described [18], in a non-invasive way and without the use of tracer dyes, in anesthetized mice using a laser Doppler perfusion image (LDPI) device (MoorLDPI-2, Moor Instruments, Axminster, Devon, UK). To direct the light beam (830 nm laser) and to avoid interference from surrounding cutaneous blood flow in the measurements, a dark plastic ring was positioned around the site of xenotransplantation. Non-manipulated (n = 8; control group) and incised but non-grafted (n = 8; non-xenografted group) animal were used as controls. During the capture of images, the ambient light level was kept at a minimum to avoid any influence on the laser light and the recorded signals. In addition, animals were kept at a constant temperature of 37°C for 5 min before and during the measurements. The mean pixel value per area of each scanned image was calculated using the software MoorLDI V5.3, and the calculated mean flux was expressed as perfusion units (PU), representing the average blood flow in the region of interest.

### Gene expression analyses

Total RNA extraction was performed in six xenografted and six non-xenografted ovarian pieces (control) using RNeasy Mini kit (Qiagen GmbH, Hilden, Germany) according to the manufacturer’s instructions. Total RNA was extracted from samples using the Rneasy Micro Kit (Qiagen GmbH), according to the manufacturer’s instructions, and treated with DNAse I (27 units for 15 min at room temperature for every sample). The RNA samples were reversely transcribed using the SuperScript III First-Strand Synthesis Supermix (Invitrogen, Carlsbad, CA, USA) according to the manufacturer’s instructions, using oligo(dT)_20_ primers, dNTP mix, Superscript III RT, RNaseOUT, MgCl_2_, RT buffer in a final volume of 20μL. The samples were first incubated at 65°C for 5 min and then at 50°C for 50 min. The reaction was finished at 85°C for 5 min and then chilled on ice. After that, RNase H was added to the samples and incubated at 37°C for 20 min. The RNA quantification and purity for each sample was performed using 1μL of sample in spectrophotometer nd-100 (Nanodrop, Wilmington, DE, USA).

Relative quantification was performed in triplicate using Real-Time PCR (ABI Prism 7300 Sequence Detection Systems, Applied Biosystem, Foster City, CA, USA). Reactions were prepared using a mixture of Power SYBR Green PCR Master Mix (Applied Biosystems), primers, nuclease-free water and cDNA. The target genes were *BAX*, *BLC-2*, *PRDX1*, *FSHR*, *IGF1R*, and *IGF2R*. The amount of RNA used was 12 ng per reaction, ranging according to the optimal concentration previously identified. Expression of *β-ACTIN* gene was used as an endogenous reference. The cDNA template was denatured at 95°C for 10 min, followed by 45 cycles at 95°C for 15s, gene-specific primer annealing temperature for 30s (Table 1) and elongation at 60°C for 30s. After each PCR run, a melting curve analysis was performed to confirm that a single specific product was generated. No-template controls, comprised of the PCR reaction mix without nucleic acid, were also run with each primer to confirm the absence of contaminations. Primer efficiency was calculated using LinReg PCR software [23] for each reaction. The primer efficiency was 1.94, 1.69, 1.97, 1.97, 1.87, 1.94 and 1.92 for *β-ACTIN*, *BAX*, *BCL-2*, *PRDX1*, *FSHR*, *IGF1R* and *IGF2R* genes. Calculations of relative quantification were performed by the REST software [24]. The gene expression patterns of the control tissue (non-xenografted) were used as calibrators in order to calculate the relative abundance of transcripts between control and xenotransplanted groups, respectively. Values are shown as n-fold difference relative to the calibrator. Additionally, the BAX/BCL2 expression ratio also was calculated. All procedures were performed as previously described [25].

**Table 1:** Primer sequences used for relative gene expression analysis by real-time polymerase chain reaction.

Gene name	Primer sequences (5’–3’)	Annealing temperature (°C)	Fragment size (bp)	GenBank accession no. or reference
*PRDX1*	F: ATGCCAGATGGTCAGTTCAAGR: CCTTGTTTCTTGGGTGTGTTG	53	224	(23)
*IGF1R*	F: CGCTGGATGTCCCCTGAGTR: GTTGTCCGGCTTGTCCAGAA	53	180	(25)
*IGF2R*	F: CAGGTCTTGCAACTGGTGTATGAR: TTGTCCAGGGAGATCAGCATG	53	137	(25)
*β-ACTIN*	F: GACATCCGCAAGGACCTCTAR: ACATCTGCTGGAAGGTGGAC	53	205	NM_173979
*BAX*	F: CCACCAAGAAGCTGAGCGAR: ACTGGTGCTCAAGGCCCTG	60	174	XM_010823819. 1
*BCL-2*	F: TGGATGACCGAGTACCTGAAR: CAGCCAGGAGAAATCAAACA	60	120	XM 586976
*FSHR*	F: GCCCCTTGTCACAACTCTATGTCR: GTTCCTCACCGTGAGGTAGATGT	60	104	NM_174061. 1

### Evaluation of *in vitro* developmental competence of xenograft-derived oocytes

Ten days after xenotransplantation, 62 grafts were recovered and the *cumulus*-oocyte complexes (COC, n = 27) were harvested using a slicing technique [26]. The xenograft-derived oocytes were morphologically classified as described elsewere [25]. The COC obtained from non-grafted ovaries (n = 102) were used as control. Groups of 20–40 COC were then placed into four-well plates (NUNC, Roskilde, Denmark) for *in vitro* maturation (IVM). Each well contained 400 μL of tissue culture medium (TCM–199, Gibco BRL) supplemented with 20 μg/mL follicle stimulating hormone (FSH; Pluset, Serono, Italy), 0.36 mmol/L sodium pyruvate, 10 μmol/L sodium bicarbonate, 50 mg/mL streptomycin/penicillin and 10% estrous cow serum. The COC were incubated for 22–24 h at 38.5°C in a humidified atmosphere containing 5% CO_2_. For *in vitro* fertilization, frozen/thawed semen (from one bull with known fertility) was centrifuged at 8,000 rpm for 7 min in a Percoll (Nutricell Nutrientes Celulares, Campinas, SP, Brazil) discontinuous density gradient (45–90%) to obtain motile sperm. The pellet was centrifuged again at 3,200 rpm for 5 min in FERT-TALP medium. At the end of *in vitro* maturation, 20–30 COCs were co-incubated for 20 h under the same atmospheric conditions with 2 × 10^6^ spermatozoa/mL in 100 μL drops of FERT-TALP medium supplemented with heparin (20 μg/mL) and bovine serum albumin (BSA) fatty acid free (6 mg/mL) under mineral oil. For embryo culture, presumptive zygotes were completely denuded in a PBS solution with 0.1% hyaluronidase and then cultured in 70 μl drops of modified CR2aa medium supplemented with 2.5% fetal calf serum (Nutricell) overlaid with mineral oil for seven days in incubator at 38.5°C under 5% CO_2_, 5% O_2_, 90% N_2_ and saturated humidity. After 72 h post-insemination (hpi) (d3) 50% of the medium (CR2aa) was renewed and the cleavage rate was assessed. Finally, the blastocyst rate was evaluated at 168 hpi (d7).

### Statistical analyses

All data were tested for normality and homoscedasticity of the variances. Parametric data were analyzed by ANOVA, and the differences were compared by Tukey’s test, whereas Student-test was used for two-parameter analysis. Relative gene expression analyses were performed with the REST software, using a pair-wise fixed reallocation randomization test and were based on primer efficiency. All data are expressed as means±SEM, and a *P*-value < 0.05 was considered as statistically significant. All the analyses were carried out using the GraphPad Prism (version 5, La Jolla, California, USA).

## Results

**Fig 1:**
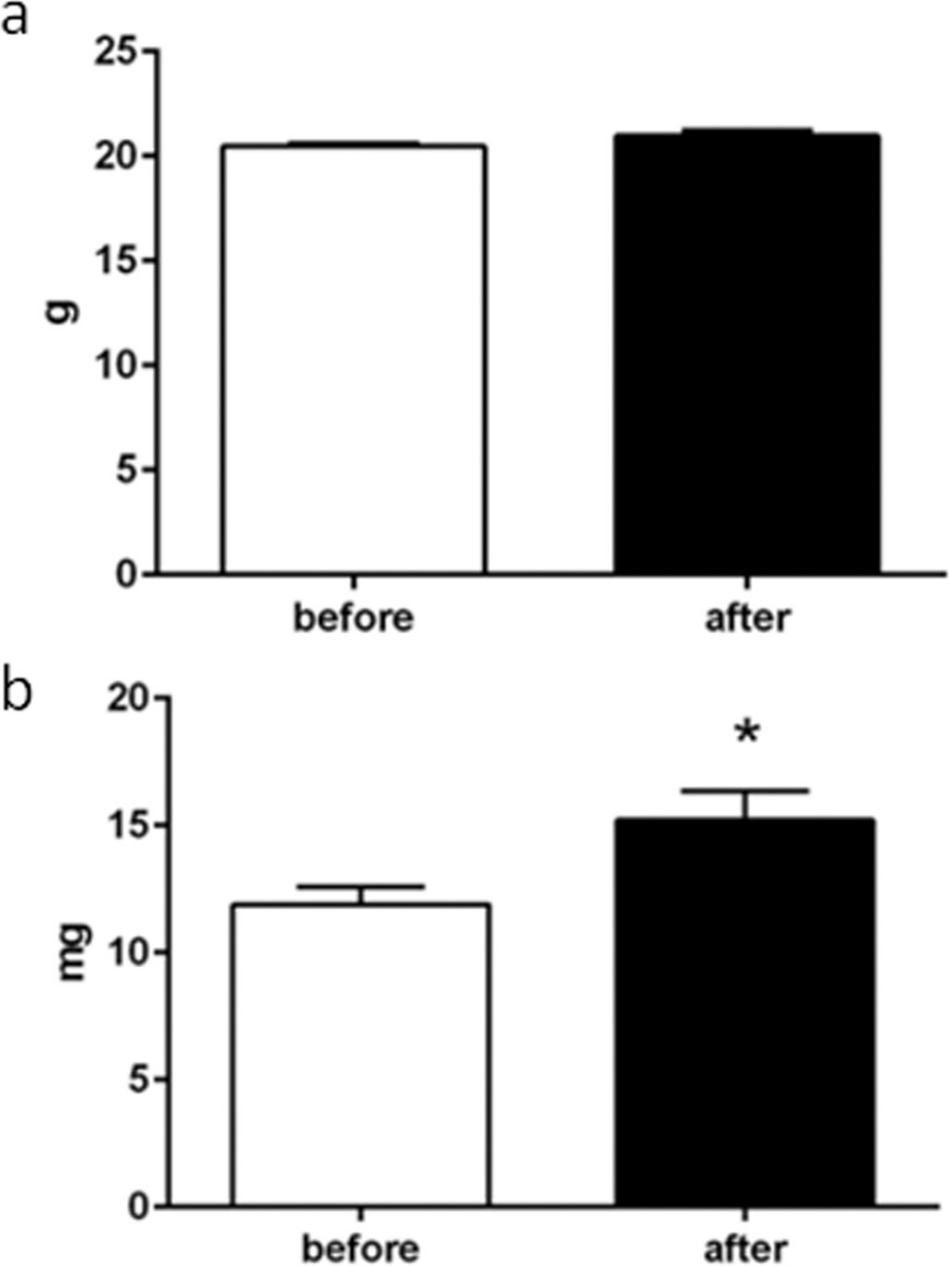
Biometric data of the mice and grafts. (a) There was no difference between host mice body weights (g) before and after xenotransplantation. However, (b) the graft weight (mg) 10 days after xenotransplantation was greater (*P<*0.01) than before.

**Fig 2:**
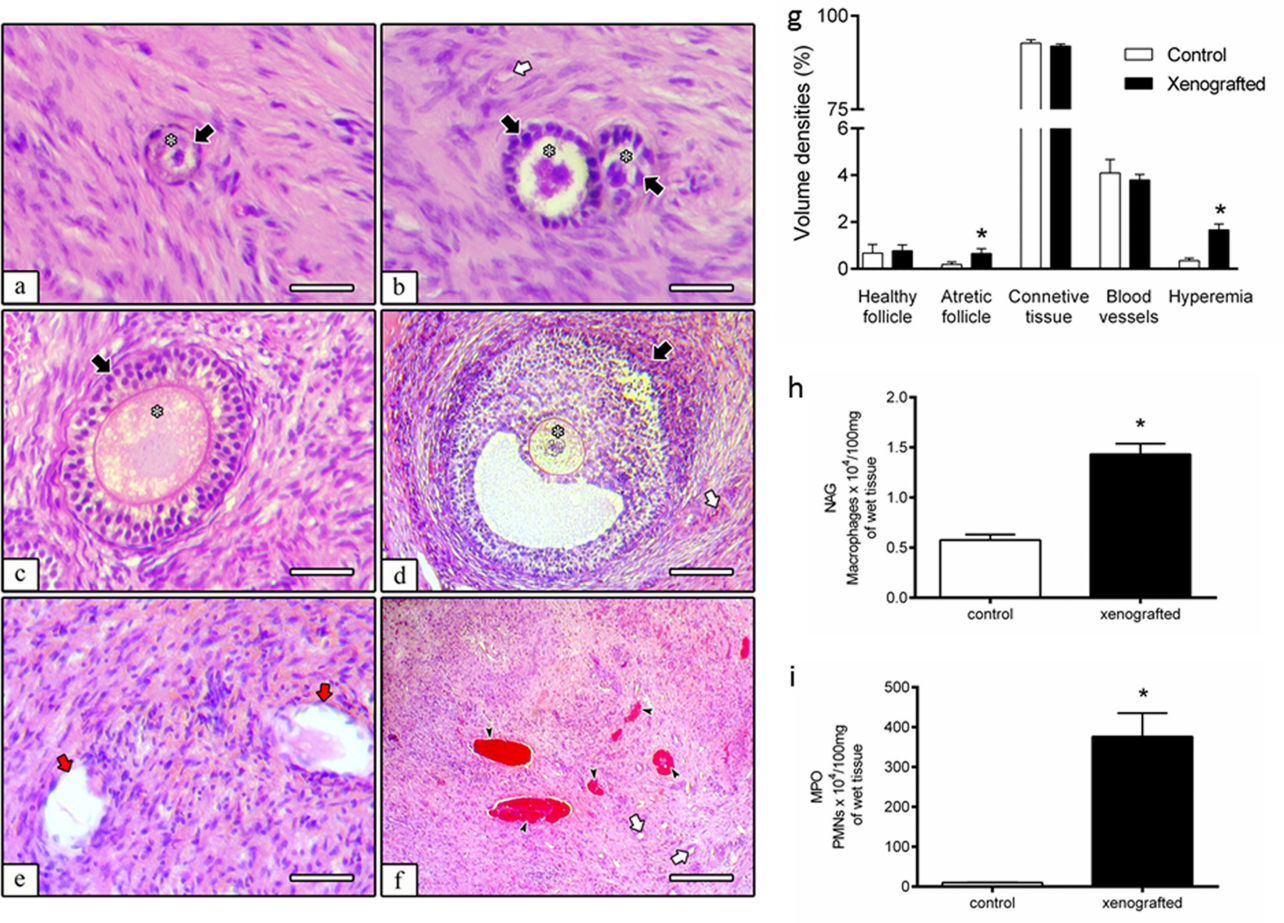
Progression of folliculogenesis, morphometry and inflammatory cell numbers in xenografted and in control ovarian fragments. (a) Primordial, (b) primary, (c) secondary, (d) antral and (e) atretic follicles were noted 10 days after xenotransplantation. (g) Volumetric density (%) of ovarian pieces before and after xenotransplantation showing a higher incidence of follicular atresia and hyperemia (f; *P<*0.01) after this procedure. (h, i) As demonstrated using (h) NAG and (i) MPO assays in xenografted ovarian wet tissue, higher concentration of host-derived macrophages and neutrophils were observed (*P<*0.01). Black arrows = healthy follicles; red arrows = atretic follicles; white arrows = blood vessels; black arrowhead = hyperemia; asterisks = oocytes. Bars in a, b, c and e = 80 μm and d and f = 150 μm.

The host body weight before and after xenotransplantation were not different (*P>*0.05), however, the grafts increased their weight (*P<*0.01; Fig 1). In histological examinations, primordial, primary, secondary, antral, and atretic follicles were identified in the grafts 10 days after xenotransplantation (Fig 2A–2E), similarly as observed in the controls (non-xenografted fragments). Connective tissue (ovarian stroma), blood vessels, and hyperemia (Fig 2A–2F) were observed in xenografts. By morphometrical analyses, the volume densities (percentages) of healthy follicles, connective tissue, and blood vessels in ovarian pieces before and after xenotransplantation were not different (*P>*0.05), however the xenografts showed a higher volumetric density of atretic follicles and hyperemia (*P<*0.01; Fig 2G). Additionally, an inflammatory response against the xenotransplant was observed, as depicted by the quantification of the total number of infiltrating tissue host-derived macrophages (*P<*0.01; Fig 2H) and neutrophils (*P<*0.01; Fig 2I), using NAG and MPO assays, respectively.

**Fig 3:**
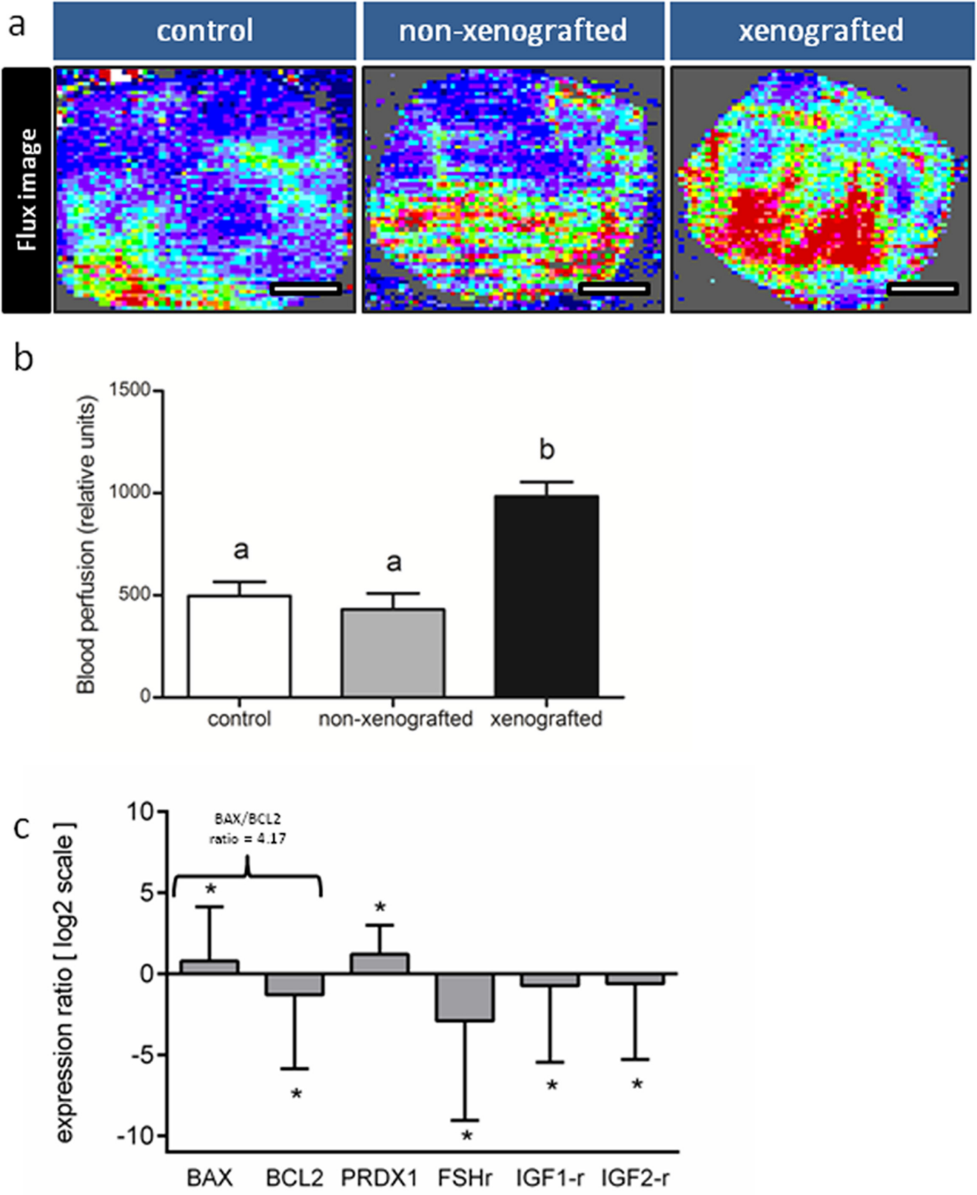
Blood perfusion and gene expression pattern in ovarian xenografts. (a, b) The back skin of xenotransplanted animals showed higher blood perfusion, when compared to non-grafted and control animals (*P<*0.01). (c) Furthermore, the analysis of gene expression, using real time PCR, showed that grafting entails in up-regulation (*P<*0.01) of genes related to apoptosis (BAX) and oxidative stress (PRDX1) whereas the genes related to follicle survival (BCL2) and progression of folliculogenesis (FSH-r, IGF1-r and IGF2-r) were down-regulated (*P<*0.01).

The blood perfusion in the back skin in transplantation sites of host animals was 128.5% and 97.9% higher than in control and non-grafted animals, respectively (*P<*0.01; Fig 3A and 3B). However, this parameter was similar between control and non-grafted groups (*P>*0.05). The genes *BAX* (1.71-fold) and *PRDX1* (2.31-fold) were up-regulated (*P<*0.01), when compared to the control group (non-xenografted), while the *BCL2* (-0.41-fold), *FSHR* (-0.13-fold), *IGF1R* (-0.6-fold), and *IGF2R* (-0.65-fold) were down regulated (*P<*0.01; Fig 3C). Moreover, the *BAX*/*BLC2* gene expression ratio in xenotransplanted ovarian pieces was 4.17 (*P<*0.01; Fig 3C).

**Fig 4:**
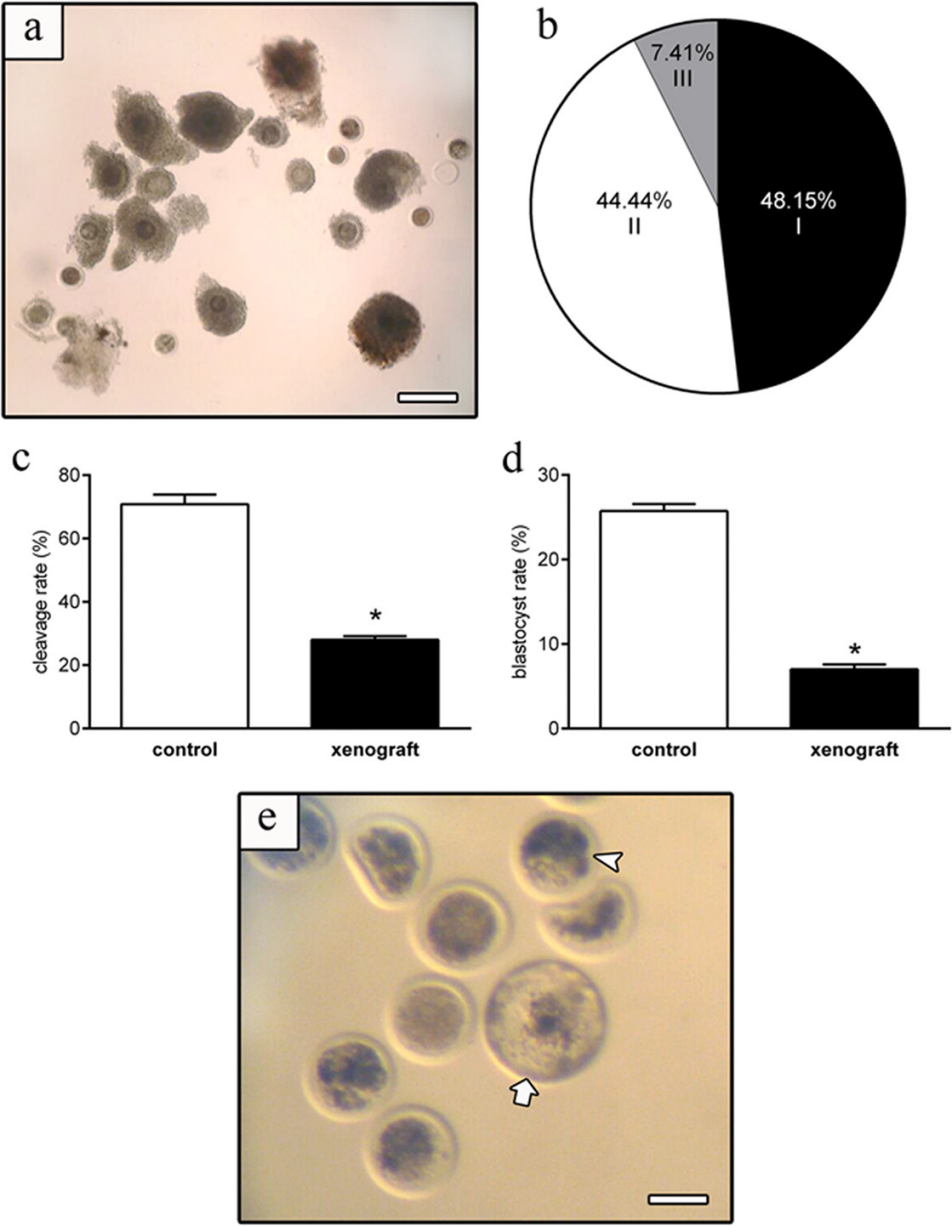
(a) Oocytes recovered from xenografts 10 days after transplantation. (b) Percentage of grade I, II and III oocytes derived from xenografts. (c) Cleavage and (d) blastocyst rates from control and xenograft groups. (e) Blastocysts (arrow), and early blastocyst (produced after *in vitro* fertilization of xenografted-derived oocytes. Bars in a = 200 μm and e = 800 μm.

Ten days after ovary xenotransplantation, 27 COCs were successfully harvested (0.43 oocytes per graft) (Fig 4A), from which 48.15% were classified as grade I, 44.44% as grade II, and 7.41% as grade III (Fig 4B). Finally, after in vitro fertilization, despite cleavage and blastocyst rates of xenograft-derived oocytes was lower than in control (*P<*0.01; Fig 4C and 4D), we observed that xenograft derived oocytes were fertile and able to give rise to blastocysts (Fig 4E).

## Discussion

Ovarian xenotransplantation is a promising alternative to preserve fertility of patients undergoing oncologic treatments, with the additional benefit of avoiding the risk of cancer cell reintroduction. However, several aspects of this procedure remained to be more accurately investigated [2]. Use of human ovaries in xenotransplantation studies is limited mainly due to ethical restrictions. The bovine ovaries have some similarities with human (as similar ovarian size, diameter of the mature follicle and of corpus luteum, follicular populations in the cortex region, as well as both being monovular and polycyclic species). Thus, it has been used as an experimental model for the study of human folliculogenesis [10, 11]. In the past decade, the applicability of ovarian xenotransplantation was investigated using bovine model, and follicular development up to the antral stage and presence of mature oocytes were reported [2]. The novelty of the present study was that for the first time we were able to demonstrate that bovine xenografted-derived oocytes can give rise to blastocysts. Furthermore, we also investigated functional aspects of xenografts (*e*.*g*. gene expression, blood perfusion, and inflammation) that were not previously addressed.

The blood supply in the site of transplantation has a crucial impact on follicle survival and development [2, 27–28]. The most commonly used xenotransplantation sites are the kidney capsule, the ovarian bursa and the intramuscular spaces [2]. These options provide a profuse blood supply to the grafts; however, they also exert a physical pressure on the grafts that may limit the expansion of growing follicles. As we successfully performed the testis xenograft under the back skin of immunodeficient mice [18], in the present study, we used this site to host ovarian pieces. As expected from the results of previous studies, ten days after xenotransplantation the ovarian pieces weight increased about 28%, and primordial, primary, secondary, and antral health follicles were observed. Although it has been classically described as a poorly vascularized area, the subcutaneous site has many advantages, including easier access by surgery, ample space for follicular development, convenience for monitoring, and easy accessibility to the follicles [2].

In longitudinal studies using subcutaneous xenotransplantation of bovine and human ovaries, a noticeable activation of primordial follicles was observed, and the subsequent follicular development required approximately 8 to 20 weeks [10, 11]. As in the present study the ovarian pieces were harvested ten days after xenotransplantation, probably the healthy antral follicles observed were derived from growing follicles activated prior to xenotransplantation. When follicles are xenotransplanted and exposed to adverse conditions (*e*.*g*. ischemia, different body temperature and immunologic rejection), follicle development and oocyte quality could be affected [12]. Thus, the higher volume density of atretic follicles observed in the present study was predictable. Previous studies suggested that ischemic conditions that occurs just after ovarian transplantation is the main factor that contributes to follicular atresia [12–14].

In the current study, the *BAX* gene was up-regulated and the *BCL2* was down-regulated in ovarian pieces after xenotransplantation, indicating a pro-apoptotic stimulus, as observed previously [14]. The B cell lymphoma-2 (BCL2) family proteins are key regulators of the apoptotic process. They are characterized by the presence of one or more conserved domains, called BH (for ‘BCL2 homology’) domains. In humans, 21 members of this family have been identified [29] and classified into three subgroups: *i*) multi-domain anti-apoptotic (e.g. BCL2, BCL-XL, and BCL2L10), *ii*) multi-domain pro-apoptotic (*e*.*g*. BAX, BAK, and BOK) and *iii*) BH3-only pro-apoptotic members (e.g. BID, BAD and BIM). These proteins exert their function at the mitochondrial level by regulating the permeability of the outer mitochondrial membrane, and the evaluation of the *BAX*/*BCL2* expression ratio have been used as a valuable indicator of the apoptosis/survival balance in ovaries.

Follicular apoptosis after transplantation has been associated with the initial ischemia of the grafts [12–14]. The mechanisms behind ischemic injury involve energy depletion and oxidative stress, which produces reactive oxygen species, such as hydroxyl radicals, superoxide anion, and hydrogen peroxide. This can eventually cause damage to lipids, DNA, enzymes and structural proteins, leading to follicular atresia [15]. The PRDX1 belongs to a six-member family of peroxidases involved in antioxidant defenses and intracellular signaling, and are present in granulosa cells [15]. In the present study the *PRDX1* gene was up-regulated in xenografts, probably as a physiological response to reduce hydrogen peroxide and alkyl hydroperoxides. Recently, some studies showed that the *PRDX1* was overexpressed in various human tumors such as breast, lung, urinary, and hepatocellular carcinoma, and its interaction with toll-like receptor 4 (TLR4) stimulates tumor angiogenesis in prostatic carcinoma [30]. As in the present study we observed an increased blood flow in the grafts, a pro-angiogenic effect of *PRDX1* overexpression also should be considered.

Furthermore, gene expression of several inflammatory factors is initiated by hypoxia-sensitive response elements, resulting in transmigration of neutrophils and macrophages [31]. During the revascularization process, vascular endothelial growth factor (VEGF) and transforming growth factor (TGF) bmRNA expression are up-regulated [12, 14, 31]. In the present study we observed an increased number of neutrophils and macrophages. As a matter of fact, is well established that these leucocytes are able to produce pro-angiogenic factors that regulate tumor survival, growth, and metastasis. For instance, macrophages produce angiogenin, CXCL8/interleukin (IL)-8 chemokine, VEGF-A and VEGF-B, while neutrophils release IL-8, VEGF-A and metalloproteases [31]. Thus, the great number of macrophages and neutrophils on xenografts could be within the increased blood flow observed.

The expression of the *FSHR* has been used as key marker of follicular developmental potential. In the present study, we observed that the *FSHR* gene was down-regulated in ovarian pieces after xenotransplantation. This finding corroborates our morphological data and the expression pattern of BAX, indicating that after grafting occurs a decrease in FSH responsiveness, which is associated to follicular atresia. Pituitary gonadotropins play a central role in follicular development, particularly in the antral phase [32]. The FSH promotes antral follicular development and stimulates the production of steroids from the granulosa cells, and consequently follicles with low FSHR concentrations become atretic [32–33].

The role of Insulin-like Growth Factors (IGFs) and their receptors in folliculogenesis has been extensively studied and they are considered essential to follicular growth, stimulating proliferation and differentiation of granulosa cells and steroidogenesis in thecal cells. The IGF1R is expressed in all classes of follicles (primordial to antral), whereas the IGF2R is expressed only in antral follicles [16]. In the current study we showed that xenotransplantation leads to down-regulation of *IGF1R* and *IGF2R* genes. *In vivo* evidences indicate that IGF1 plays a key role in the responsiveness of the ovary to FSH action, so the decrease in IGF1 responsiveness in xenografts probably results in a FSHR down-regulation, leading to an increase in follicular atresia. In despite of the similarities between human and bovine ovaries, there are some differences, for example, cow ovaries contain more fat tissue. Maybe this could also influence the expression of IGF-1/2receptor, perfusion and inflammatory response.

In the present study, we were able to produce blastocysts *in vitro* from oocytes harvested from xenografts of cow’s ovaries. The ability to reach blastocyst stage is one of the best approaches to evaluate the oocyte developmental potential, as in this stage the embryo has passed through the maternal-zygote transition and is dependent on the expression of its own genome. Although live pups were previously produced from mice ovary grafts transplanted under kidney capsule of nude rats [9], in cattle no embryos from xenografted-derived oocytes reached blastocyst stage [11]. The lower cleavage and blastocyst rates observed in the present study for the xenograft-derived oocytes, when compared to the controls, probably reflects the sub-optimal conditions of follicular development related to the functional changes in the grafts. Nevertheless, it remains valid as a proof of concept, and further studies may address alternative strategies to improve graft function (e.g., with exogenous gonadotrophic support) and increase *in vitro* embryo production rates.

Taking together, our findings shows that, in spite of functional changes related to inflammation and abnormal gene expression patterns that may lead to poor FSH and IGF responsiveness and pro-apoptotic balance, oocytes recovered from follicles growth in xenografted ovarian fragments are able to give rise to blastocysts. These findings demonstrate that short term ovarian xenotransplant is an alternative assisted reproductive technology and may be used to preserve the female fertility. In association to cryopreservation of ovarian fragments, short term xenotransplants can also overcome the limitations of the long term approach for patients undergoing complex chemotherapy protocols.
